# Supplementary material for: The role of disulfide bonds in a Solanum tuberosum saposin-like protein investigated using molecular dynamics
Source: PLoS One. 2020 Aug 25;15(8):e0237884. doi: 10.1371/journal.pone.0237884 (PMC7447066; doi:10.1371/journal.pone.0237884)
Supplement: S1 Table — (DOCX) [file pone.0237884.s001.docx]

S1 Table: Normalized dimer variation

| **Sample Code** | **pH 3.0** | | | **pH 7.4** | | |
| --- | --- | --- | --- | --- | --- | --- |
|  | **Dimer** | **Helical^‡^** | **Loop^†^** | **Dimer** | **Helical^‡^** | **Loop^†^** |
| **AI** | 0.0418 ± 0.0047 | 0.0452 ± 0.0035 | 0.0306 ± 0.0091 | 0.0481 ± 0.0012 | 0.0484 ± 0.0016 | 0.0469 ± 0.0021 |
| **D1** | 0.0372 ± 0.0007 | 0.0434 ± 0.0036 | 0.0170 ± 0.0091 | 0.0523 ± 0.0035 | 0.0583 ± 0.0070 | 0.0324 ± 0.0087 |
| **D2** | 0.0414 ± 0.0042 | 0.0449 ± 0.0033 | 0.0299 ± 0.0075 | 0.0453 ± 0.0042 | 0.0547 ± 0.0051 | 0.0141 ± 0.0041 |
| **D3** | 0.0460 ± 0.0020 | 0.0476 ± 0.0019 | 0.0407 ± 0.0064 | 0.0484 ± 0.0016 | 0.0544 ± 0.0010 | 0.0287 ± 0.0090 |
| **D4** | 0.0405 ± 0.0051 | 0.0459 ± 0.0072 | 0.0228 ± 0.0035 | 0.0517 ± 0.0052 | 0.0573 ± 0.0102 | 0.0331 ± 0.0119 |
| **D5** | 0.0482 ± 0.0038 | 0.0522 ± 0.0012 | 0.0348 ± 0.0175 | 0.0503 ± 0.0068 | 0.0481 ± 0.0031 | 0.0575 ± 0.0195 |
| **D6** | 0.0484 ± 0.0020 | 0.0552 ± 0.0055 | 0.0260 ± 0.0120 | 0.0455 ± 0.0031 | 0.0529 ± 0.0042 | 0.0213 ± 0.0007 |
| **AR** | 0.0409 ± 0.0022 | 0.0454 ± 0.0014 | 0.0262 ± 0.0057 | 0.0472 ± 0.0017 | 0.0544 ± 0.0025 | 0.0234 ± 0.0094 |

Monomer A and Monomer B were summed across their residues and normalized to the number of residues in the appropriate group (Dimer: 206, Helical: 158, Loop: 48). The average and standard deviation of the three replicates is reported.

^‡^Helical residues exclude D40 to G63

^†^Loop residues are D40 to G63 inclusive
