# Supplementary figures and images for: The role of disulfide bonds in a Solanum tuberosum saposin-like protein investigated using molecular dynamics
Source: PLoS One. 2020 Aug 25;15(8):e0237884. doi: 10.1371/journal.pone.0237884 (PMC7447066; doi:10.1371/journal.pone.0237884)

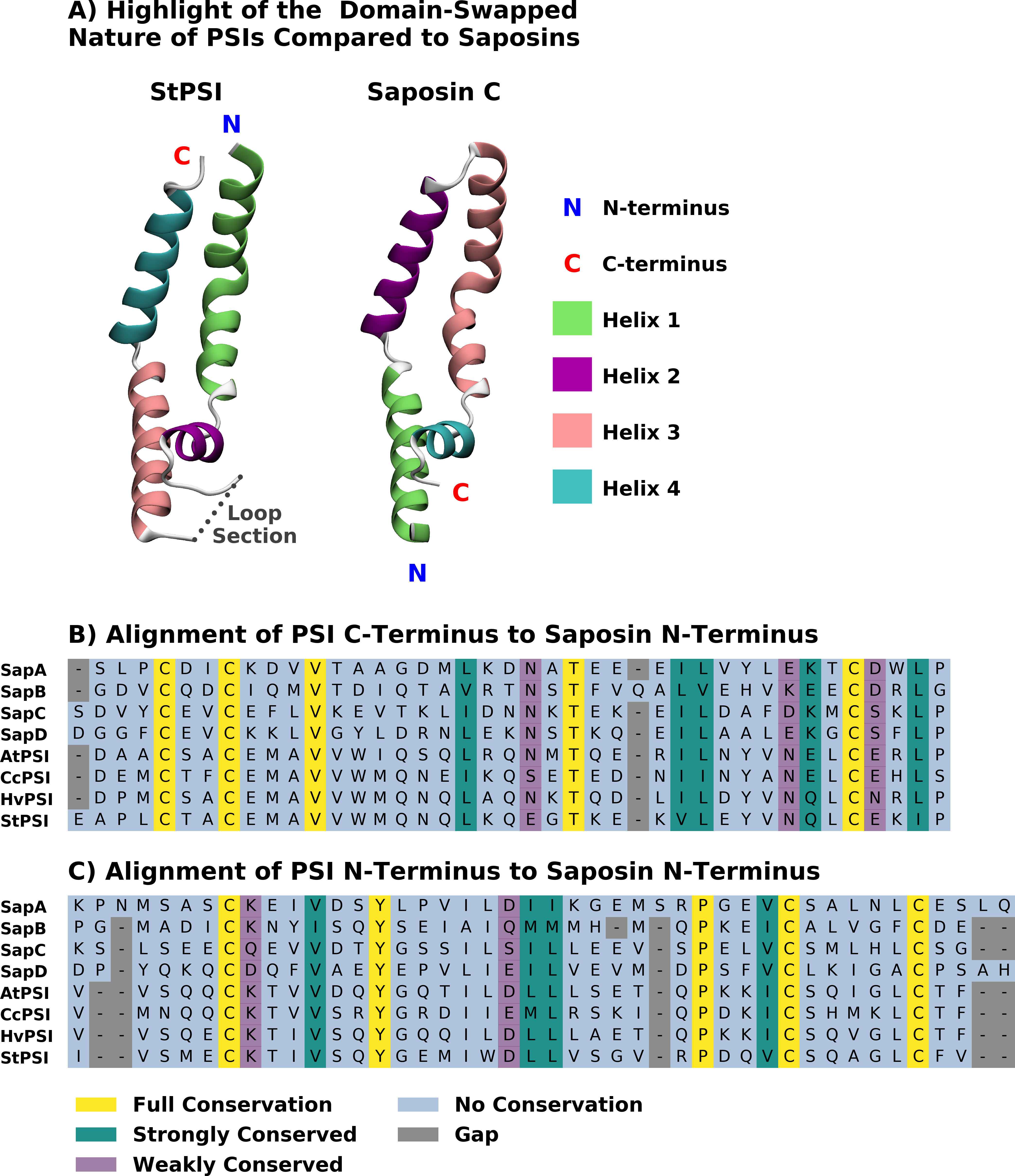

Supplement: S1 Fig — Panel A) Structural alignment of the Solanum tuberosum PSI (StPSI) (PDB ID: 3RFI) and saposin C (PDB ID: 2QYP). Note: The unresolved loop section in the StPSI was omitted in this alignment and rendering. Coloring is as per upper legend for panel A. Panel B) Clustal-Omega alignment of the C-terminal half of four PSIs with the N-terminal half of saposins A-D. Coloring is as per bottom legend. SapA–saposin A; SapB–saposin B; SapC–saposin C; SapD–saposin D; AtPSI–Arabidopsis thaliana PSI; CcPSI–Cynara cardunculus PSI; HvPSI–Hordeum vulgare PSI. The loop portion of the PSIs used was omitted from the alignment. Panel C) Clustal-Omega alignment of the N-terminal half of four PSIs with the C-terminal half of saposins A-D. Details are as per Panel B. (TIFF) [file pone.0237884.s002.tiff]

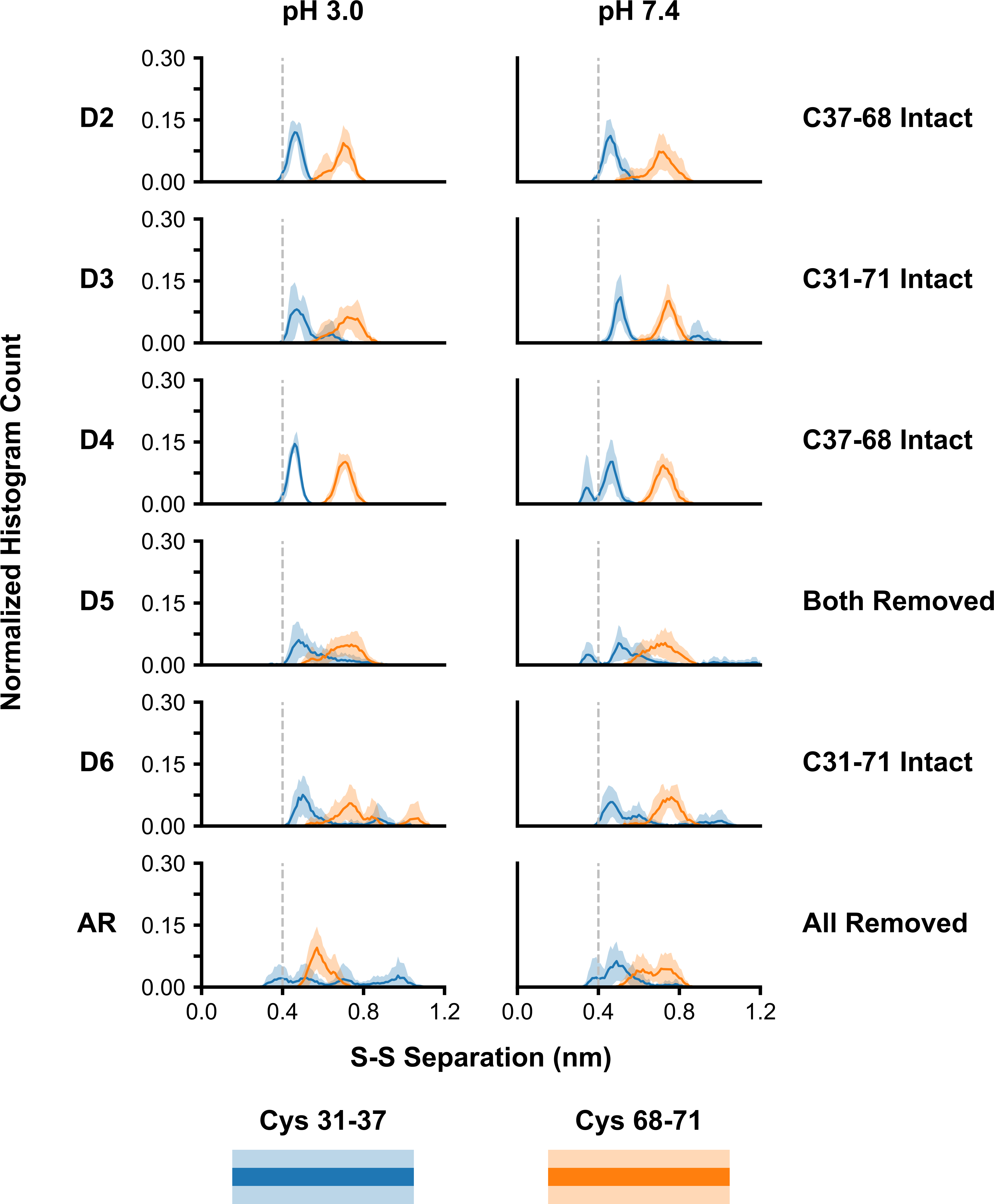

Supplement: S2 Fig — The pairs examined are between C31-C37 (blue), and C68-C71 (orange). Data were histogrammed over the last 10% of the simulations for each monomer and replicate. Dark lines are the average per-bin across the replicates and monomeric units (n = 6). Lighter shaded regions correspond to the per-bin standard deviation. The grey dashed line denotes 0.4 nm, the cutoff used in this paper to define a possibility of disulfide interchange occurring. (TIFF) [file pone.0237884.s003.tiff]

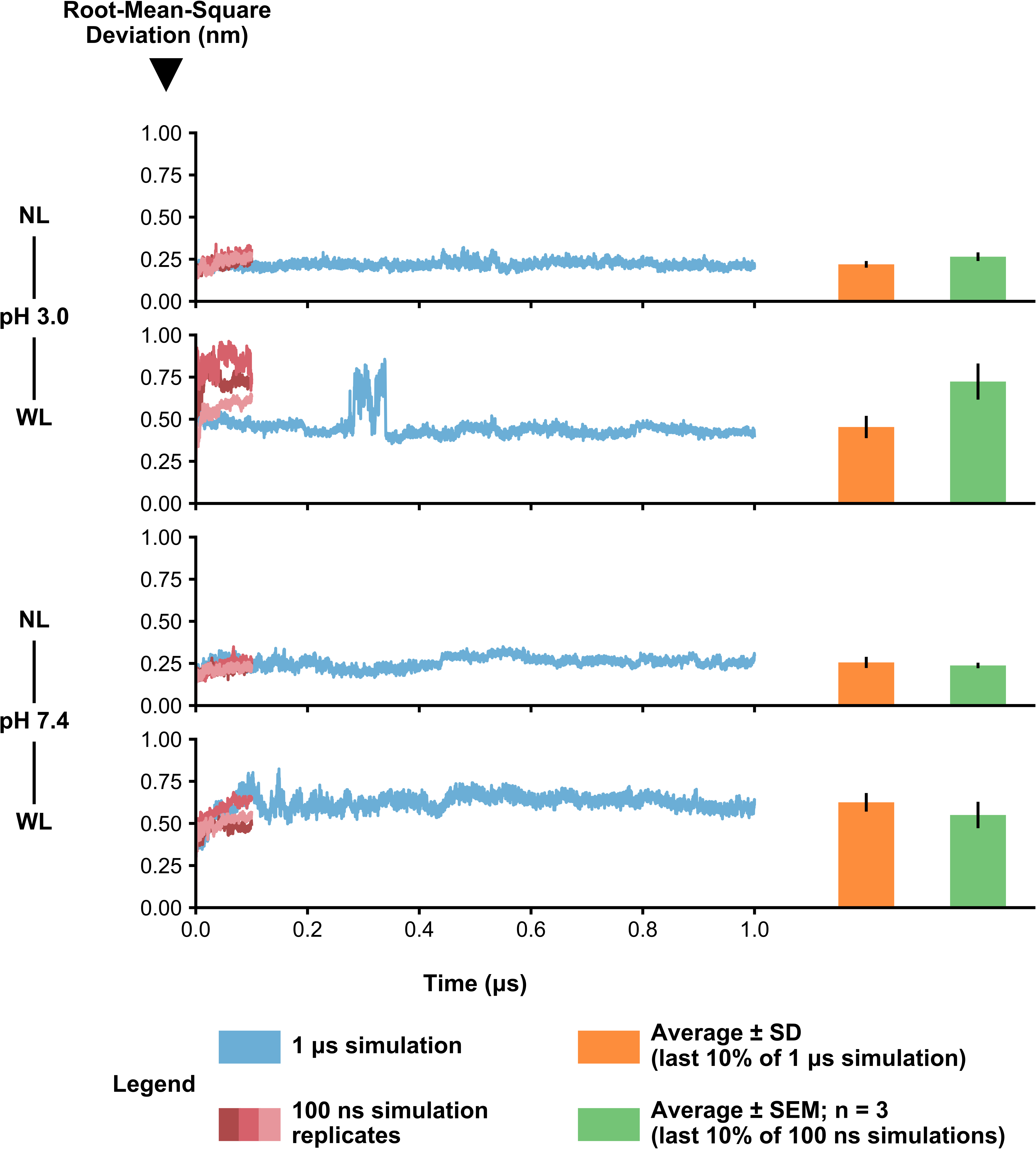

Supplement: S3 Fig — pH 3.0 –top; pH 7.4 –bottom. Red shades– 100 ns AR simulations (); blue– 1 μs AR simulation. NL–loop residues 40 to 63 omitted for the calculation; WL–loop residues included for the calculation. Bar graphs on right are the average ± standard deviation or standard error of the mean (where appropriate) over the last 10% of the simulation trajectory/trajectories. (TIFF) [file pone.0237884.s004.tiff]

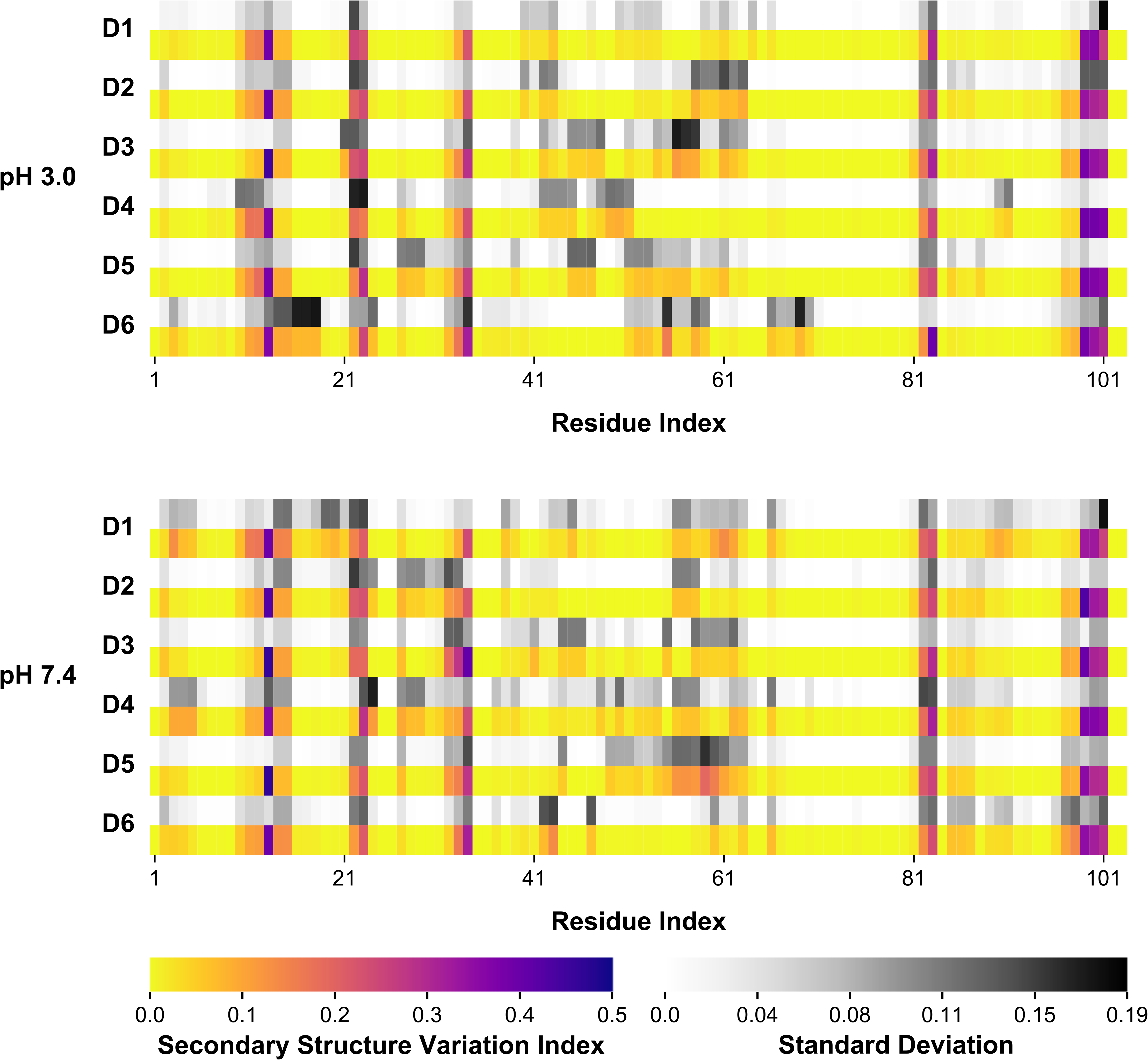

Supplement: S4 Fig — pH 3.0 –top; pH 7.4 –bottom. For sample codes, see Table 1. The color of the cells is proportional to the average normalized variation per residue. The standard deviation is presented immediately above the presented variation on a separate color scale. (TIFF) [file pone.0237884.s005.tiff]

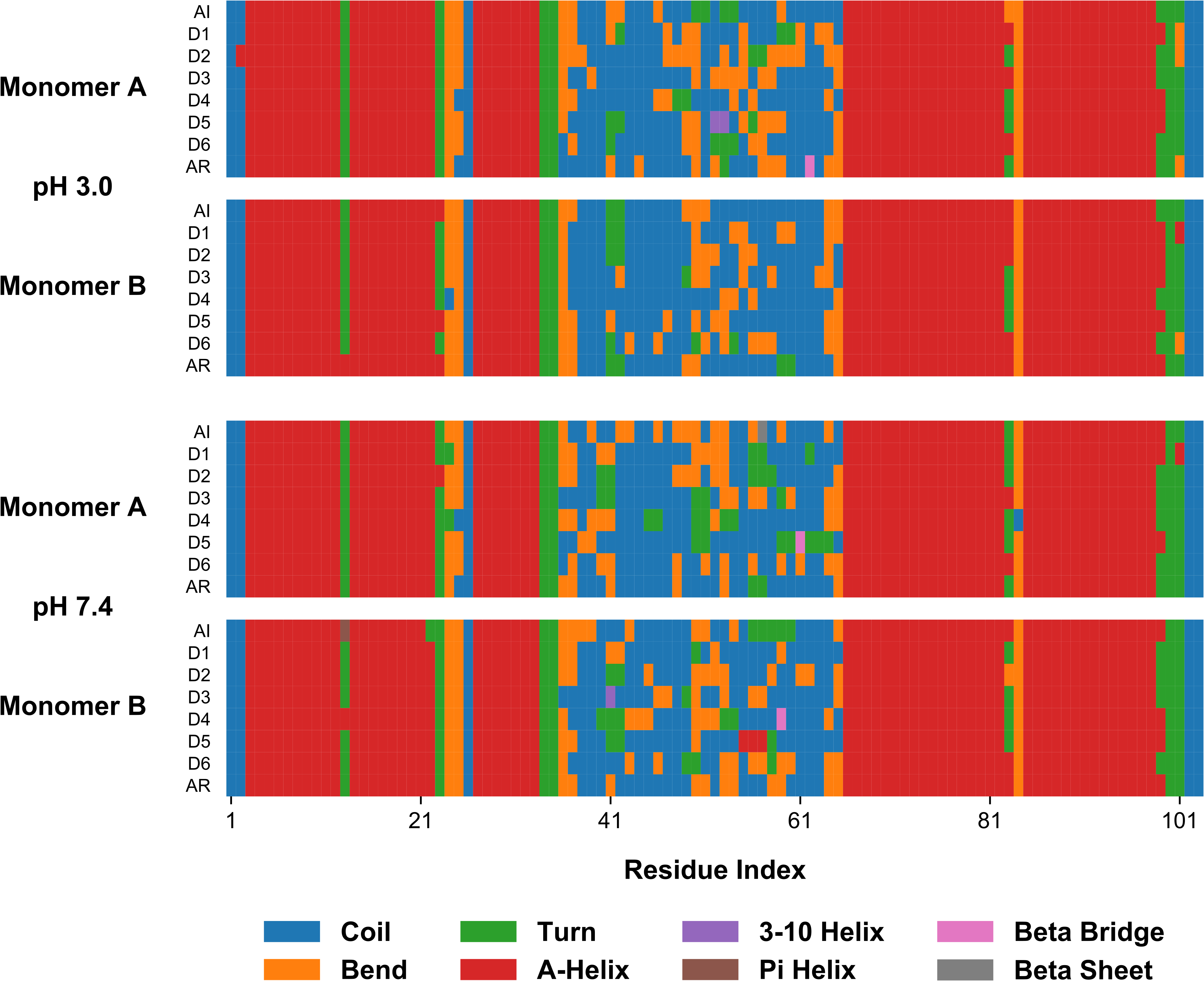

Supplement: S5 Fig — pH 3.0 –top; pH 7.4 –bottom. The color of the cells represents the mode of the calculated secondary structure on a residue basis. (TIFF) [file pone.0237884.s006.tiff]

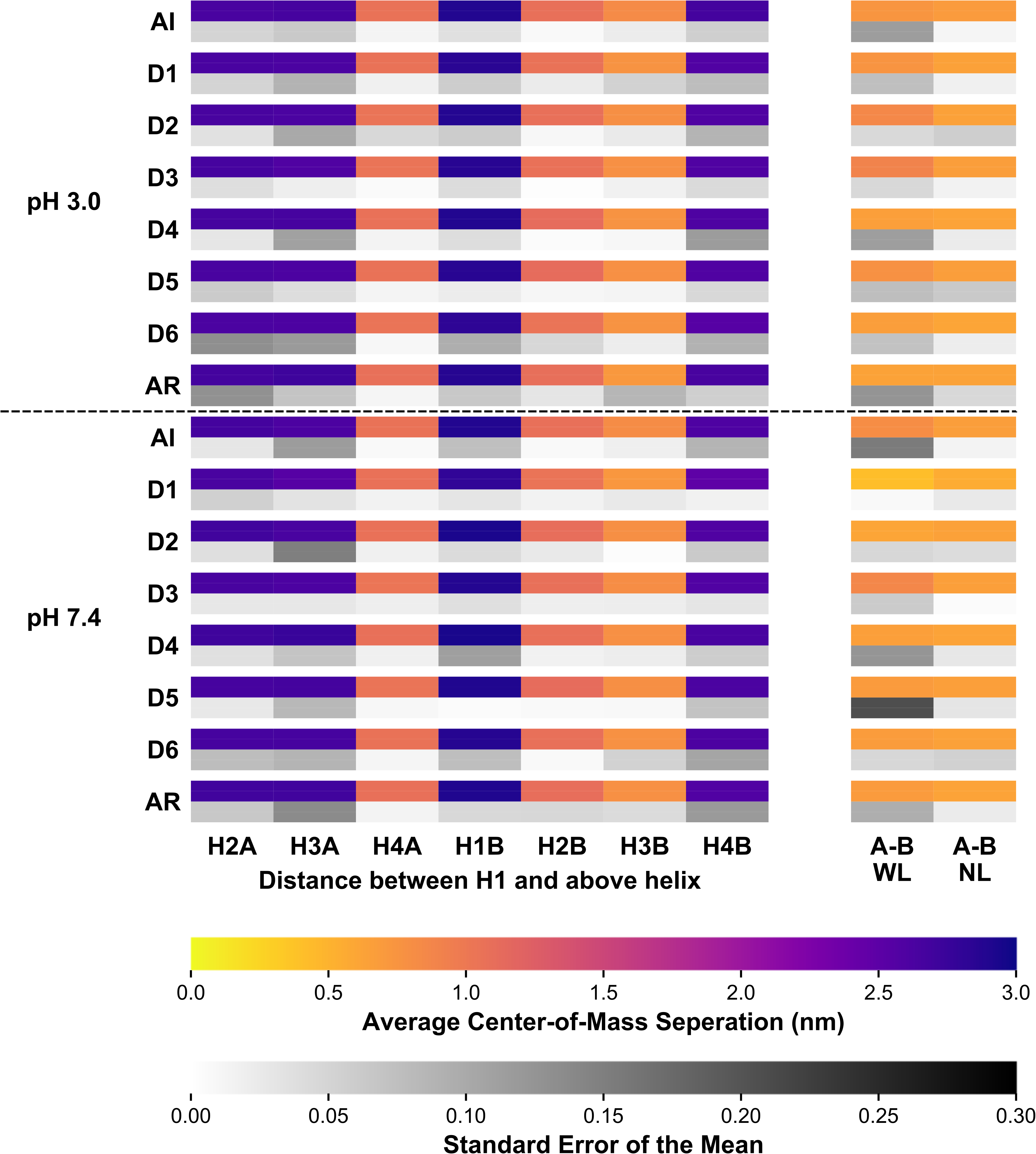

Supplement: S6 Fig — Left: The separation of helices relative to helix 1 are presented for example purposes. Right: The separation of monomer A and monomer B, with the loop portion (WL) or with no-loop (NL) portion included in the calculation. (TIFF) [file pone.0237884.s007.tiff]

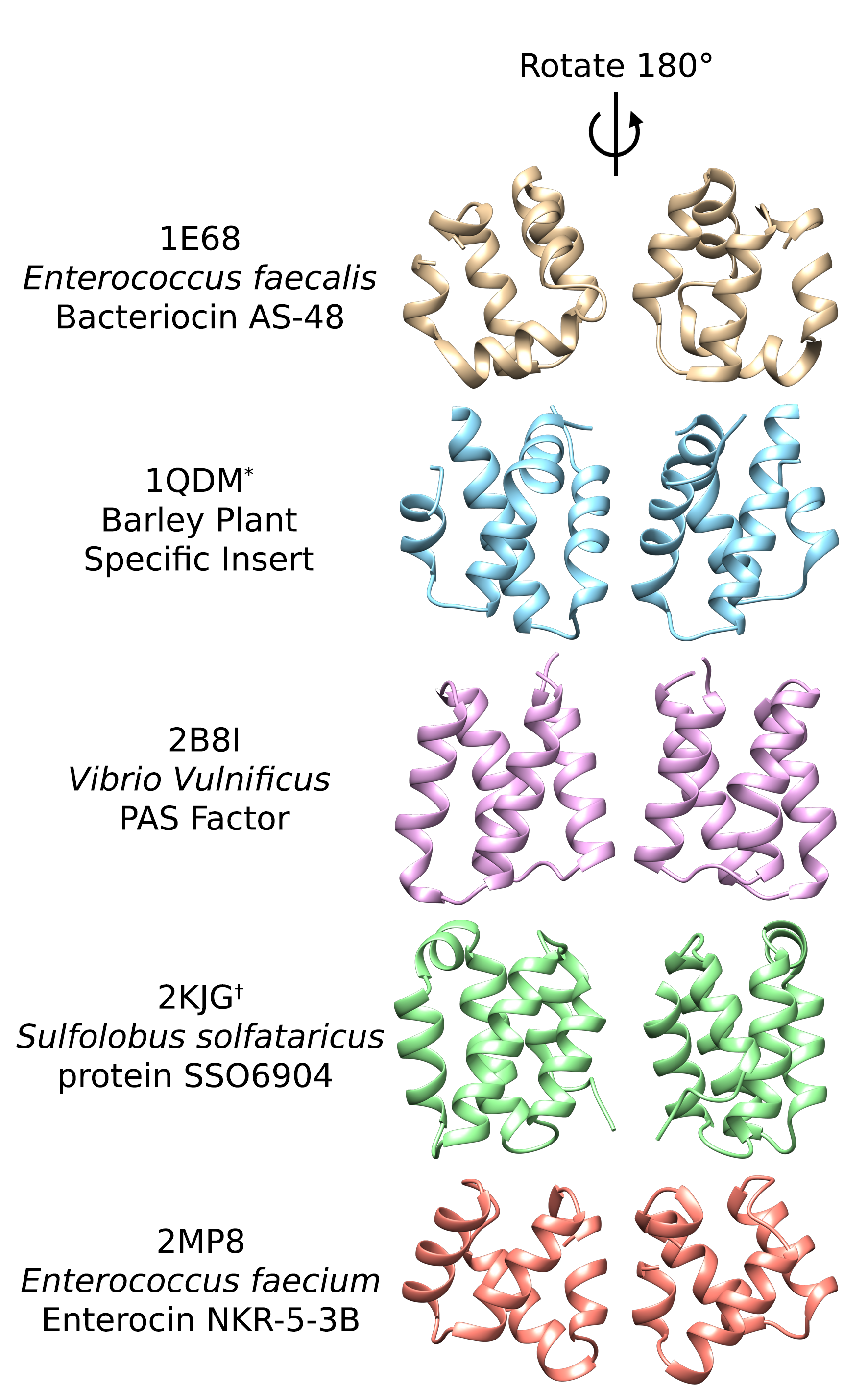

Supplement: S7 Fig — PDB codes for each structure are given in the figure. If more than one model was present in the PDB file, only the first one model was used; *Loop residues Cys320 to Asp349 were omitted from the figure for clarity; †N-terminal residues Lys91 to His99 were omitted from the figure for clarity. (TIFF) [file pone.0237884.s008.tiff]
